# Impact of concomitant vasoactive treatment and mechanical left ventricular unloading in a porcine model of profound cardiogenic shock

**DOI:** 10.1186/s13054-020-2816-8

**Published:** 2020-03-18

**Authors:** Nanna L. J. Udesen, Ole K. L. Helgestad, Ann B. S. Banke, Peter H. Frederiksen, Jakob Josiassen, Lisette O. Jensen, Henrik Schmidt, Elazer R. Edelman, Brian Y. Chang, Hanne B. Ravn, Jacob E. Møller

**Affiliations:** 1 Department of Cardiology, Odense University Hospital, J. B. Winsløwsvej 4, Odense C, Denmark; 2 Department of Cardiology, Rigshospitalet, Copenhagen University Hospital, Copenhagen, Denmark; 3 Department of Anesthesiology and Intensive Care, Odense University Hospital, Odense, Denmark; 4 Institute for Medical Engineering and Science, Massachusetts Institute of Technology, Cambridge, Massachusetts USA; 5 Cardiovascular Division, Brigham and Women’s Hospital, Harvard Medical School, Boston, Massachusetts USA; 6 Department of Cardiothoracic Anaesthesia, Rigshospitalet, Copenhagen University Hospital, Copenhagen, Denmark

**Keywords:** Cardiogenic shock, Acute myocardial infarction, Vasopressor, Mechanical circulatory support, Cardiac work, Organ perfusion

## Abstract

**Background:**

Concomitant vasoactive drugs are often required to maintain adequate perfusion pressure in patients with acute myocardial infarction (AMI) and cardiogenic shock (CS) receiving hemodynamic support with an axial flow pump (Impella CP).

**Objective:**

To compare the effect of equipotent dosages of epinephrine, dopamine, norepinephrine, and phenylephrine on cardiac work and end-organ perfusion in a porcine model of profound ischemic CS supported with an Impella CP.

**Methods:**

CS was induced in 10 pigs by stepwise intracoronary injection of polyvinyl microspheres. Hemodynamic support with Impella CP was initiated followed by blinded crossover to vasoactive treatment with norepinephrine (0.10 μg/kg/min), epinephrine (0.10 μg/kg/min), or dopamine (10 μg/kg/min) for 30 min each. At the end of the study, phenylephrine (10 μg/kg/min) was administered for 20 min. The primary outcome was cardiac workload, a product of pressure-volume area (PVA) and heart rate (HR), measured using the conductance catheter technique. End-organ perfusion was assessed by measuring venous oxygen saturation from the pulmonary artery (SvO_2_), jugular bulb, and renal vein. Treatment effects were evaluated using multilevel mixed-effects linear regression.

**Results:**

All catecholamines significantly increased LV stroke work and cardiac work, dopamine to the greatest extend by 341.8 × 10^3^ (mmHg × mL)/min [95% CI (174.1, 509.5), *p* < 0.0001], and SvO_2_ significantly improved during all catecholamines. Phenylephrine, a vasoconstrictor, caused a significant increase in cardiac work by 437.8 × 10^3^ (mmHg × mL)/min [95% CI (297.9, 577.6), *p* < 0.0001] due to increase in potential energy (*p* = 0.001), but no significant change in LV stroke work. Also, phenylephrine tended to decrease SvO_2_ (*p* = 0.063) and increased arterial lactate levels (*p* = 0.002).

**Conclusion:**

Catecholamines increased end-organ perfusion at the expense of increased cardiac work, most by dopamine. However, phenylephrine increased cardiac work with no increase in end-organ perfusion.

## Introduction

Reversing the vicious cycle of critically low cardiac output (CO) and perfusion pressure in acute myocardial infarction and cardiogenic shock (AMICS) remains challenging, leading to sustained mortality of approximately 50% for the last few decades [1–5]. Hemodynamic support strategies aim to restore central perfusion using vasoactive drugs as first-line therapy [6], followed by the use of mechanical circulatory support devices [7, 8]. Exogenous catecholamines stimulating α- and/or β-adrenergic receptors are administrated in about 90% of AMICS cases [1, 9]. However, observational studies suggest that high dosage and prolonged use of vasoactive drugs are associated with increased mortality [10, 11]. β-Adrenergic agonists improve inotropic and chronotropic state, thus improving CO by enhancing myocardial contractility and increasing heart rate (HR) [12]. Although the use of vasoactive drugs to increase perfusion pressure and flow is theoretically beneficial in AMICS, it comes at the expense of increased left ventricular (LV) mechanical work with the potential to accelerate myocardial ischemia and induce arrhythmias [6]. Vasoconstriction without a concomitant increase in CO may also aggravate organ hypoperfusion [13]. Mechanical circulatory support devices seem appealing and are increasingly used in AMICS to overcome the potential adverse effects and limitations of catecholamines [1, 14]. The Impella CP is a transvalvular axial flow pump with the inlet placed in the LV and the outlet in the ascending aorta and is capable of pumping up to 3.5 L/min oxygenated blood from the LV to the aorta. The forward flow increases systemic and coronary perfusion while reducing cardiac work [15]. Despite Impella support, additional pharmacological support is often necessary to maintain adequate perfusion pressure [10, 16], and the optimal vasoactive drug choice is currently unknown.

Thus, this study aimed to compare cardiac work and end-organ perfusion during infusion of equipotent dosages of four commonly used vasoactive agents (epinephrine, dopamine, norepinephrine, and phenylephrine) in pigs with experimentally induced CS supported by the Impella CP device.

## Methods

### Animals

Ten female Danish Landrace pigs weighing approximately 70 kg were studied. The study was approved and conducted per guidelines of the Danish Animal Experiments Expectorate (authorization number: 2016-15-00951). Unfractionated heparin (20 IU) was administered every 2 h to avoid blood clotting during the experiment. Amiodarone (300 mg) was injected before instrumentation followed by continuous infusion of 50 mg/h to avoid malignant arrhythmias. Instrumentation was done using the percutaneous Seldinger technique, except for the surgical exposure of the internal jugular vein. Instrumentation included placement of a conductance catheter (Ventri-Cath 512 PV Loop Catheter, Millar Inc.) in the LV for continuous recordings of pressure-volume (PV) relationships, a conductance catheter in the aorta to measure aortic pressure, a central line, and a continuous CO 7.5-Fr Swan-Ganz catheter with SvO_2_ recording (Edwards Lifesciences Corp. Irvine, CA, USA) placed in the pulmonary artery. A conventional triple lumen 7-Fr Swan-Ganz catheter (Edwards Lifesciences Corp. Irvine, CA, USA) was placed in the renal vein via femoral venous access, and a 4-Fr double-lumen central venous catheter (Cook Medical, Bloomington, USA) in a retrograde fashion in the internal jugular vein to obtain organ-specific blood gasses for measuring oxygen saturation and lactate levels.

### Experimental protocol

Before the start of the study, a sealed envelope listing the order of the infusions of epinephrine, norepinephrine, and dopamine was handed to an independent individual who prepared and labeled the infusions with a number signifying the order. The infusions were prepared and administered at fixed infusion rates to what was considered equipotent doses and not to target a predefined MAP, equivalent to a dose of norepinephrine 0.10 μg/kg/min, dopamine 10 μg/kg/min, epinephrine 0.10 μg/kg/min, and phenylephrine 10 μg/kg/min. The infusion of phenylephrine alone was not blinded given the long half-life and administered in all pigs in the end. All animals were treated with a fluid regime of 1 L of isotonic saline the first hour and afterwards 900 mL/h, which was shifted between Ringer acetate and isotonic saline.

CS was induced by stepwise injection of polyvinyl alcohol microspheres (Contour™, Boston Scientific, Marlborough, MA, USA) in the left main coronary artery through a JL3.5 guide catheter (Launcher, Medtronic Inc., Minneapolis, MN, USA) [17]. Hemodynamics were allowed to stabilize for 2–3 min after each injection before the administration of the next injection. Stepwise injections of microspheres was continued until CS developed, defined as mixed venous oxygen saturation (SvO_2_) reduction to < 30% or ≤ 50% of baseline value and/or sustained cardiac index < 1.5 L/min/m^2^ for ≥10 min. A median of 12 microsphere injections (interquartile range, 9–17) was required to induce CS. Following the onset of CS, Impella CP was advanced from the left femoral artery and placed across the aortic valve with the inlet in the left ventricle and outlet in the ascending aorta. The placement of Impella CP was guided by fluoroscopy, and the maximum pump speed possible was achieved and maintained during the entire study. Vasoactive treatment with norepinephrine, dopamine, or adrenaline was randomized, and the treating team was blinded to the treating order. The first vasoactive infusion started following 30 min of Impella CP support, and each infusion was administered for 30 min. The initial experimental plan included an Impella CP only phase (no vasoactive drugs) for 30 mins and a washout phase (no infusion of vasoactive drugs) between each drug infusion. However, due to severe hypotension (mean arterial blood pressure (MAP) < 50 mmHg) during the Impella alone and washout phase in the pilot pigs, the experimental protocol was changed and Impella support was combined with a minimum dose of norepinephrine to maintain MAP > 50 mmHg. Also, the withdrawal and initiation of subsequent drug infusion overlapped to avoid any drop in arterial pressure. Vasoactive drug infusion was withdrawn following any change in hemodynamics (mean arterial pressure or heart rate). Phenylephrine was administered in all pigs for 20 min after the completion of all three catecholamine infusions, followed by euthanization.

## Data collection and analysis

### Pressure volume parameters

A conductance catheter was inserted through a sheath in the right carotid artery and advanced retrograde into the LV and connected to an MPVS Ultra® Pressure-Volume (PV) loop system (Millar Inc., 6001 Gulf Fwy, Houston, TX, USA). The PV relationships were available in 9 pigs at all time points and not available in 1 pig due to disturbance in the volume signal. The MPVS Ultra® PV loop system was connected to a PowerLab 16/35 (ADInstruments, Dunedin, New Zealand), and PV measurements were continuously recorded in LabChart Pro (ADInstruments, Dunedin, New Zealand). Volumes were calibrated using an alpha correctional value, and parallel wall conductance was determined using the hypertonic saline method. Data recorded from the conductance catheter comprised of the following: pressure-volume area (PVA, mmHg × mL), LV end-diastolic pressure (LVEDP, mmHg), LV end-diastolic volume (LVEDV, mL), LV end-systolic pressure (ESP), LV stroke work (SW, mmHg × mL), LV end-systolic pressure-volume relationship (Ees), and heart rate (HR, bpm). In all the pigs, balloon occlusion of the inferior vena cava was performed in the healthy condition at the start of the study, and the acquired *V*_0_ (theoretical ventricular volume when no pressure is generated) was kept as a constant throughout the study to generate single-beat estimations of Ees and PVA [18, 19]. Ees was derived from Ees = LVESP/(LVESV-V0) [20]. Potential energy (mmHg × mL) was estimated using the formula, PE = LVESP(LVESV-V0)/2 [21]. All other variables were extracted from the software program. The slope of the line from LVEDV to LVESP on the P-V loop was used to calculate arterial elastance (Ea). Ventriculo-arterial coupling was assessed as the ratio between Ea and Ees [22].

### Data collection

Data were collected at seven prespecified time points: baseline before injection of microspheres, the onset of CS before initiation of Impella support, after 30 min of Impella support, at the end of each blinded infusion, and after 20 min of phenylephrine infusion. Collected data included systemic and pulmonary artery blood pressure, central venous blood pressure, blood gasses assessing oxygen saturation and lactate levels from the femoral and pulmonary artery, and renal and internal jugular veins. PV relationships including LVEDP, LVEDV, LVESP, LVEDP, SW, potential energy, PVA, HR, Ees, and Ea were determined for the same time points.

### Efficacy parameters

The primary efficacy parameters of the study were PVA and cardiac work (HR × PVA), both parameters closely related to myocardial oxygen consumption [21]. End-organ perfusion was estimated based on organ-specific (cerebral and renal) and overall venous saturations.

## Statistical analysis

Baseline variables are presented as mean (95% confidence interval (CI)). A linear mixed model (LMM) was constructed using individual pigs as subjects for random factors and sequential experimental stages as fixed repeated measurements. The LMM allowed the correlation between subjects and non-constant variability over time. It was used to calculate the change in a variable following an intervention compared to the control once the normal distribution of the variables residual was confirmed. A sensitivity analysis was conducted for an outlier to assess its effect on the overall interpretation of the results. All statistical tests were performed using STATA 15. A *p* value < 0.05 was considered significant.

## Results

### Hemodynamic changes during CS and after 30 min of Impella support

**Fig. 1:**
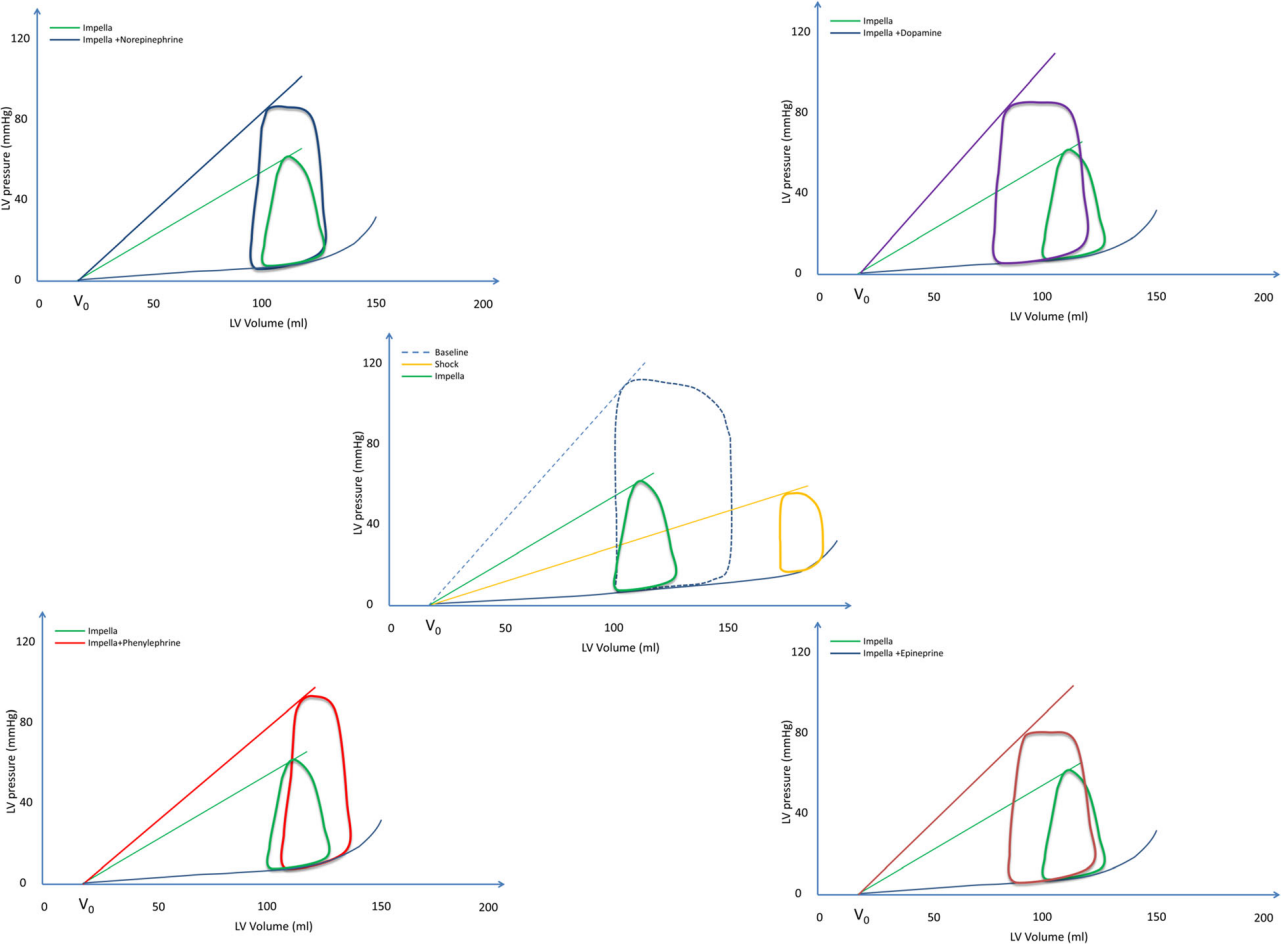
Representative pressure-volume loops during therapeutic interventions. The PV loop at baseline, cardiogenic shock, and during Impella is depicted in the middle panel. The PV loop depicting the effect of concomitant vasoactive agent and Impella compared to Impella alone is depicted in the side panels. The PV loops represents the average effects on each intervention

Following the induction of CS, defined as a 50% reduction in SvO_2_ and cardiac index < 1.5 L/min/m^2^, a significant increase in LVEDV and LVEDP was observed concomitant with a significant reduction in SW and stroke volume (Table 1 and Fig. 1). The systemic blood pressure was compromised with a mean MAP of < 40 mmHg, Table 1.

**Table 1:** Hemodynamic characteristics and organ perfusion at baseline, onset of cardiogenic shock, and after 30 min of Impella CP support

Variable	Baseline	Cardiogenic shock	Impella 30 min
	Mean (95% CI)	Mean (95% CI)	Mean (95% CI)
Stroke work (mmHg × mL)	4853 (3906, 5801)	703 (350, 1056)	980 (619, 1340)
Potential energy^2^ (mmHg × mL)	4290 (2887, 5694)	4567 (2973, 6161)	2690 (2019, 3361)
PVA^1^ (mmHg × mL)	9143 (7406, 10,880)	5270 (3468, 7072)	3670 (2780, 4560)
Heart rate (min^−1^)	82 (75, 89)	81 (75, 87)	75 (69, 82)
LV work^2^ 10^3^ × (mmHg × mL)/min	751.7 (595.2, 906.1)	430.3 (271.3, 589.2)	274.2 (206.9, 341.5)
Ees^3^ (mmHg/mL)	1.42 (1.06, 1.79)	0.40 (0.3, 0.5)	0.74 (0.57, 0.92)
LVEDV^4^ (mL)	156.1 (133, 179)	188.8 (154, 224)	125.2 (108, 142)
LVEDP^5^ (mmHg)	16 (12.7, 19.2)	22 (19.7, 24.8)	19 (15, 22)
LVESV^6^ (mL)	91.1 (69, 113.2)	162.7 (130.2, 195.2)	97 (82.4, 111.5)
LVESP^7^ (mmHg)	104.87 (91.4, 118.3)	58.35 (48.8, 67.9)	61.65 (50.5, 72.9)
Mean arterial pressure, (mmHg)	79 (71, 88)	39 (30, 48)	61 (51, 72)
Right atrial pressure (mmHg)	9 (7, 11)	14 (10, 17)	12 (9, 14)
Mean pulmonary arterial pressure (mmHg)	21 (19, 24)	25 (21, 29)	24 (20, 28)
Arterial lactate (mmol/L)	1.49 (1.05, 1.94)	2.1 (1.67, 2.51)	2.22 (1.65, 2.79)
Mixed venous oxygen saturation (%)	76 (69, 82)	37 (30, 44)	55 (47, 64)
Renal venous oxygen saturation (%)	89 (85, 92)	58 (42, 75)	73 (62, 85)
Cerebral venous oxygen saturation (%)	79 (73, 85)	45 (35, 55)	59 (48, 71)
Hemoglobin (mmol/L)	6.4 (5.7, 7.1)	5.6 (5.1, 6.2)	6.1 (5.6, 6.6)

Data are presented as mean and (95% CI). *Abbreviations*: *PVA*^*1*^ pressure volume area, *LV work*^*2*^ left ventricular work equals heart rate × pressure volume area, *Ees*^*3*^ left ventricular elastance, *LVEDV*^*4*^ left ventricular end-diastolic volume, *LVEDP*^*5*^ left ventricular end-diastolic pressure, *LVESV*^*6*^ left ventricular end-systolic volume, *LVESP*^*7*^ left ventricular end-systolic pressure

**Fig. 2:**
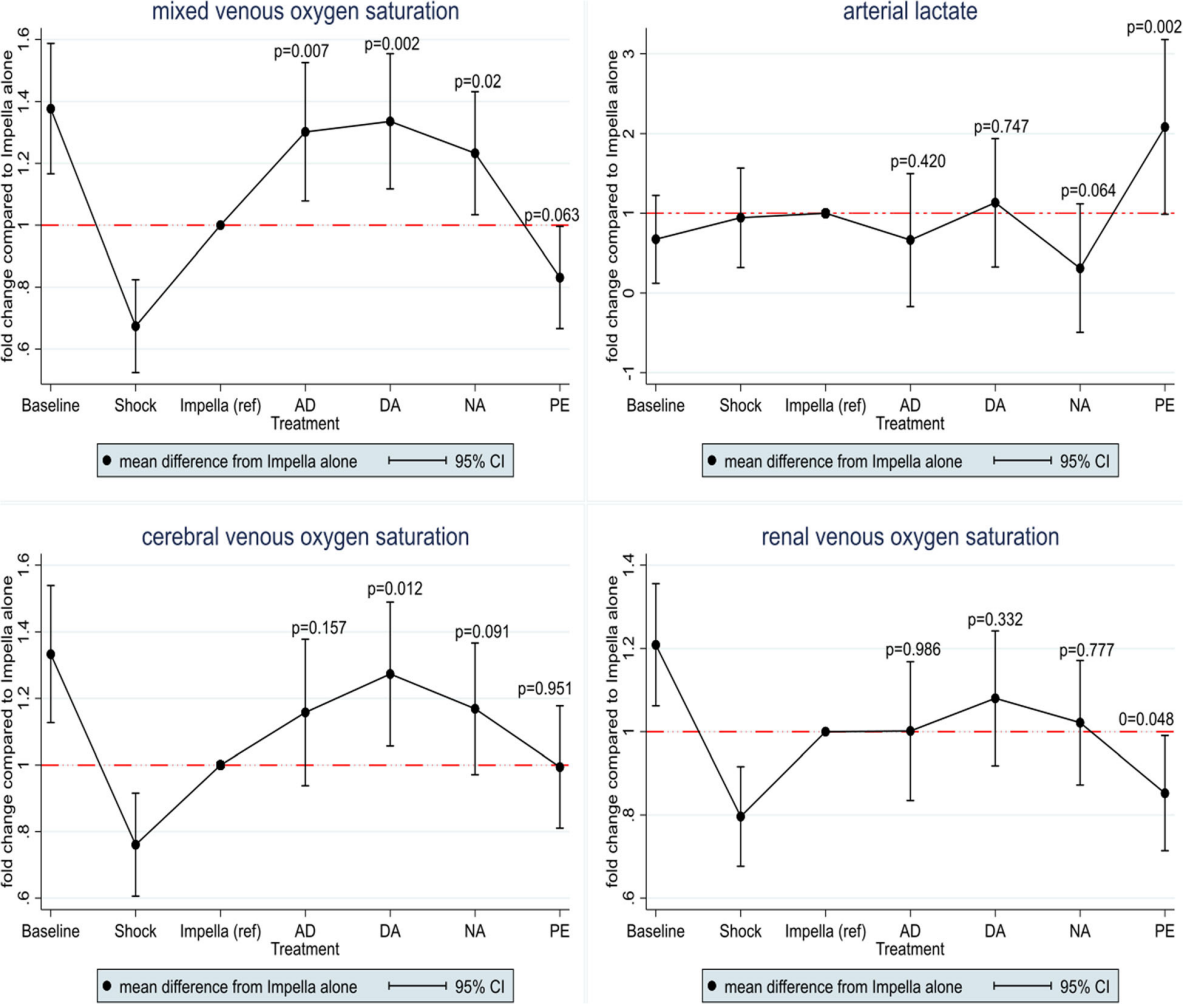
End-organ perfusion. The relative mean difference during interventions compared to Impella alone. Impella alone is represented by the red dotted line. AD, epinephrine; DA, dopamine; NA, norepinephrine; PE, phenylephrine

Initiation of Impella CP resulted in a reduction of LVEDV by 33% and potential energy by > 40% with little change in SW (Table 1). The PV loop shifted leftward and became triangular (Fig. 1). Despite Impella support, the MAP remained < 50 mmHg in 7 pigs. Hence, a low-dose norepinephrine (median 0.02 μg/kg/min [interquartile range 0.02, 0.05]) was administered to increase MAP > 50 mmHg. Impella support reduced the cardiac work (HR × PVA) via a reduction in both potential energy and HR (Table 1 and Fig. 1). Although Impella support increased renal, cerebral, and mixed venous saturations, the levels did not reach the baseline level, while the lactate levels did not change significantly during Impella support (Table 1 and Fig. 2). The SvO_2_ increased during Impella support from 37% (95% CI 39, 44) to 55% (95% CI 47, 64). A laminar aortic flow was observed after 30 mins of Impella support in four pigs, suggesting uncoupling between the ventricular and aortic peak pressure.

### Effect of vasoactive agents on cardiac workload during Impella support

All vasoactive drugs caused a significant increase in PVA, albeit to different degrees. SW increased significantly with all catecholamines, but remained unchanged with phenylephrine. On the other hand, only phenylephrine caused a significant increase in potential energy. HR increased with all drugs, except for norepinephrine. Thus, cardiac work increased significantly with all vasoactive drugs (Table 2). The average effects on PV loops are summarized in Fig. 1. In general, catecholamines increased SW and reduced LVESV, thus causing a leftward shift of the PV loop. In contrast, phenylephrine caused a rightward shift of the PV loop with an increase in LVEDV and LVEDP (Fig. 1 and Table 2).

**Table 2:** Mixed model values listed as mean difference from the reference time. The reference time was set to 30 min after initiation of Impella CP support

	Epinephrine	Dopamine	Norepinephrine	Phenylephrine
	Mean difference (95% CI)	Mean difference (95% CI)	Mean difference (95% CI)	Mean difference (95% CI)
Stroke work (mmHg × mL)	1175 (279, 2070) *p* = 0.01	1974 (1111, 2838) *p* < 0.0001	1086 (260, 1912) *p* = 0.01	604 (− 116, 1324) *p* = 0.1
Potential energy (mmHg × mL)	324 (− 1016, 1664) *p* = 0.636	− 53 (− 1346, 1239) *p* = 0.936	929 (− 306, 2165) *p* = 0.140	2220 (1142, 3298) *p* < 0.0001
PVA (mmHg × mL)	1506 (121, 2890) *p* = 0.033	1928 (593, 3263) *p* = 0.005	2023 (746, 3299) *p* = 0.002	2824 (1711, 3938) *p* < 0.0001
Heart rate (BPM)	15 (3, 28) *p* = 0.016	29 (17, 41) *p* < 0.0001	3 (−8, 15) *p* = 0.554	32 (22, 42) *p* < 0.0001
LV work 10^3^ × (mmHg × mL)/min	202.2 (28.3, 376.1) *p* = 0.023	341.8 (174.1, 509.5) *p* < 0.0001	186.3 (26, 346.6) *p* = 0.023	437.8 (297.9, 577.6) *p* < 0.0001
LVEDP (mmHg)	3 (− 0.5, 7) *p* = 0.086	0 (− 3.4, 3.8) *p* = 0.903	1 (−2.4, 4.4) *p* = 0.571	6 (2.6, 8.5) *p* < 0.0001
LVEDV (mL)	− 8 (− 30, 15) *p* = 0.510	− 15 (− 37, 7) *p* = 0.175	0 (− 21, 21) *p* = 0.978	18 (0.2, 37) *p* = 0.047
LVESP (mmHg)	23 (7.1, 39.6) *p* = 0.005	29 (13.6, 44.8) *p* < 0.0001	28 (12.7, 42.6) *p* < 0.0001	34 (21.3, 47.4) *p* < 0.0001
LVESV (mL)	− 23 (− 48, 2) *p* = 0.074	− 36 (− 60, − 12) *p* = 0.003	−10 (− 33, 12) *p* = 0.371	14 (− 6, 34) *p* = 0.170
Es (mmHg/mL)	0.75 (− 0.15, 1.65) *p* = 0.101	1.75 (0.87, 2.62) *p* < 0.0001	0.42 (− 0.41, 1.25) *p* = 0.324	0.29 (− 0.44, 1.01) *p* = 0.78
Ea (mmHg/mL)	− 0.17 (− 1.64, 1.3) *p* = 0.823	− 0.29 (− 1.71, 1.13) *p* = 0.687	0.57 (− 0.79, 1.92) *p* = 0.411	1.52 (0.34, 2.7) *p* = 0.012
Ea/Ees ratio	− 2.3 (− 4.92, 0.33) *p* = 0.086	− 2.81 (− 5.34, − 0.28) *p* = 0.029	− 1.36 (− 3.78, 1.06) *P* = 0.270	1.13 (− 0.99, 4.23) *p* = 0.296
Mean arterial pressure (mmHg)	10 (− 3.9, 23.9) *p* = 0.157	20 (6.4, 33) *p* = 0.004	13 (0.8, 25.7) *p* = 0.037	6 (− 5.1, 17.5) *p* = 0.282
Right atrial pressure (mmHg)	− 2 (− 3.6, 0.2) *p* = 0.082	−3 (− 5, − 1.3) *p* = 0.001	− 2 (− 3.7, − 0.3) *p* = 0.024	2 (0.4, 3.5) *p* = 0.016
Mean pulmonary artery pressure (mmHg)	− 2 (− 7.3, 4.3) *p* = 0.616	2 (− 3.7, 7.5) *p* = 0.513	1 (− 3.7, 6.1) *p* = 0.586	9 (4.7, 14.1) *p* < 0.0001
Hemoglobin (mmol/L)	0.64 (− 0.16, 1.45) *p* = 0.115	0.52 (− 0.26, 1.3) *p* = 0.193	0 (− 0.63, 0.81) *p* = 0.806	1.2 (− 0.51, 1.9) *p* = 0.001

For abbreviations, see Table 1. Mixed model values listed as mean difference from reference time with 95% CI. The reference time was set to 30 min after initiation of Impella CP support. A value of *P* < 0.05 was considered significant

Also, phenylephrine significantly increased right atrial and mean pulmonary artery pressure, an effect not observed with any of the other vasoactive drugs (Table 2).

### Effect of vasoactive agents on end-organ perfusion during Impella support

**Fig. 3:**
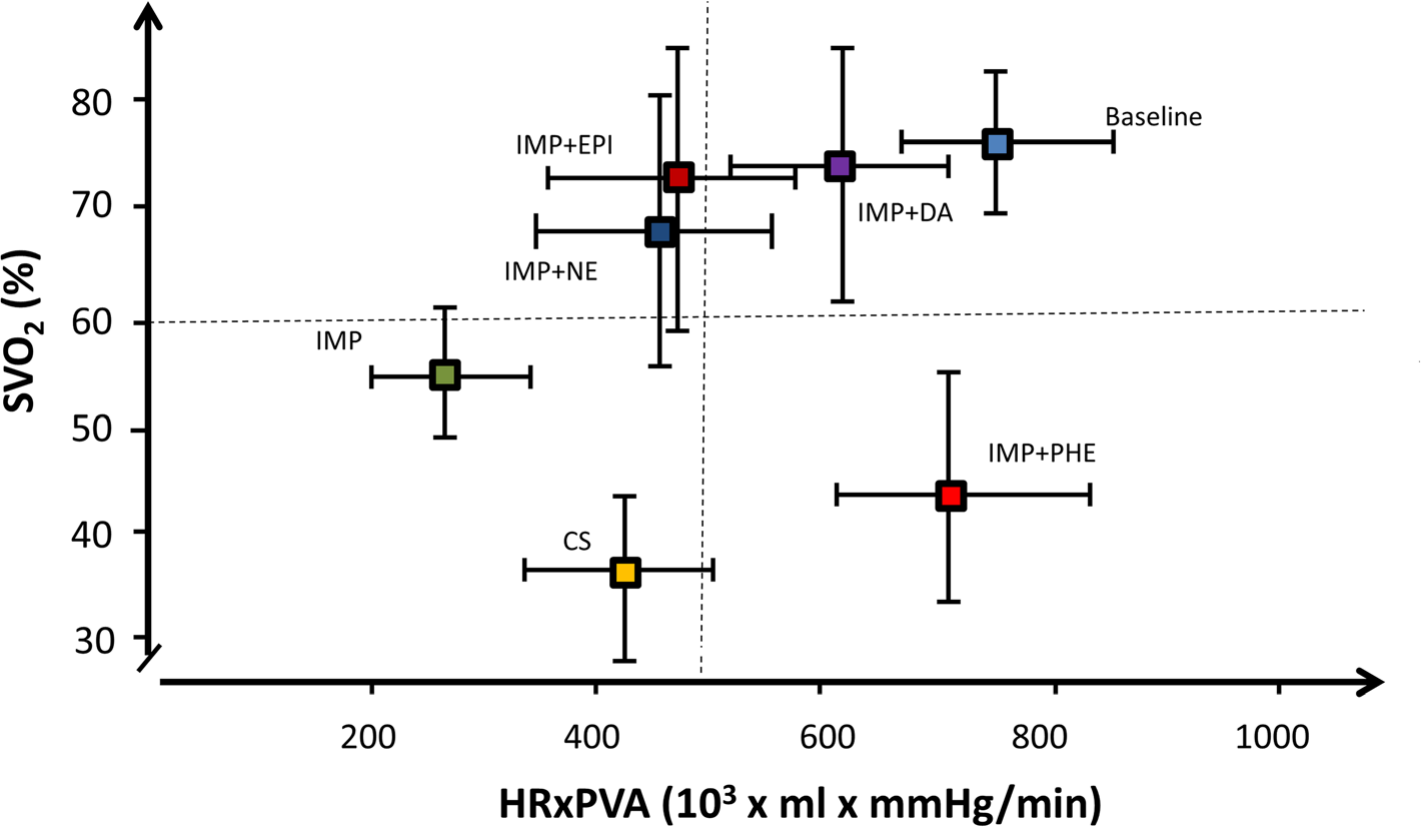
Correlation of oxygen delivery with cardiac work. SvO_2_ (*y*-axis) represents the mean mixed venous oxygen saturation and HR × PVA (*x*-axis) represents the mean cardiac work

SvO_2_ increased with vasoactive drugs, except phenylephrine which caused a slight decrease [(mean difference from Impella alone), − 9% [95% CI (− 19% to 0%)], *p* = 0.063] (Fig. 2). Renal venous saturations also decreased with phenylephrine while there was no change with any of the other drugs. Cerebral venous saturation increased significantly with dopamine, slightly with norepinephrine, and did not change with phenylephrine (Fig. 2). Signs of end-organ ischemia were observed with phenylephrine with a significant increase in arterial and venous lactate levels (Fig. 2). In contrast, the catecholamines did not cause any change in lactate levels, although there was a trend towards lower lactate concentration with norepinephrine (*p* = 0.06) (Fig. 2). The correlation between SvO_2_ and cardiac work during different stages of the study is shown in Fig. 3.

### Effect of vasoactive agents on ventricular-arterial coupling during Impella support

CS was characterized by a decrease in Ees and an increase in ventriculo-arterial decoupling (Ea/Ees) from 1.3 (1.3–2.0) to 7.2 (3.8–10.6) (Table 1). Dopamine significantly increased Ees, but no significant changes were observed with all the other drugs. Phenylephrine significantly increased Ea (Table 2). Consequently, ventriculo-arterial coupling significantly improved with dopamine (*p* = 0.03), a trend towards improvement was observed with epinephrine (*p* = 0.09), but remained unchanged with phenylephrine and norepinephrine.

## Discussion

To our knowledge, this is the first study to compare the effect of equipotent doses of commonly used vasoactive agents in combination with a microaxial flow pump. Despite the increase in oxygen delivery with lowering of cardiac workload after initiation of Impella support, perfusion pressure was not restored in the majority of pigs, which is why additional vasoactive therapy seems unavoidable. The addition of a catecholamine increased LVESP and SvO_2_ but at the expense of increased cardiac work (most for dopamine). However, vasoconstriction with phenylephrine caused an increase in cardiac work without any increase in oxygen delivery (decreased SvO_2_ and increased arterial lactate). Thus, a support strategy based on Impella CP and low-dose catecholamine (norepinephrine) seems optimal to balance oxygen delivery and LV unloading.

Patients with AMICS have critically low blood pressures, which may aggravate tissue hypoxia and cause decreased coronary blood flow even in the non-infarcted myocardium if the vicious cycle is not interrupted [7]. Impella is used in AMICS to support the flow of oxygenated blood via continuous forward flow from the LV to the aorta, thereby augmenting CO while unloading the LV [14]. In this study, initiation of Impella support decreased cardiac work through a reduction in LV potential energy, and the PV loop shape changed to triangular (Fig. 1). Despite Impella support, the mean arterial pressure remained < 50 mmHg in 7 of 10 pigs and oxygen delivery was although improved not restored to pre shock level (Fig. 3). However, increased blood pressure does not automatically translate into an increased oxygen delivery [23] as an increase in MAP can be obtained by increasing CO or vascular resistance via vasoconstriction [13]. Vasoactive agents can increase perfusion pressure by either stimulating cardiac β-adrenoceptors, thus enhancing CO or by stimulating vascular α-adrenoceptors, causing an increase in systemic vascular resistance [24]. Since phenylephrine only stimulates the α-adrenoceptors [25], it caused a high afterload and a significant increase in potential energy (energy wasting) with a rightward shift of the PV loop (Fig. 1) accompanied by reduced oxygen delivery and increased arterial lactate levels (Fig. 2). It is likely that the shift in preload, afterload, and increase in HR also led to compromised coronary blood flow. Overall, catecholamines improved oxygen delivery via an increase in both perfusion pressure and flow, but the associated LV energy costs varied among the different drugs. Particularly, dopamine increased cardiac work via an increase in both HR and PVA. The present study suggests that the optimal balance between maximum oxygen delivery and the least expense in cardiac work is achieved with norepinephrine where HR increased least (Fig. 3) and the beneficial effect is in accordance with other experimental findings [26].

The finding from this study that the Impella alone was insufficient to increase MAP and SvO2 to pre shock values is in line with observational studies reporting frequent use of concomitant vasoactive agents with Impella support in CS [10, 27–29]. Thus, vasoactive agents are often unavoidable for the treatment of AMICS supported by Impella CP, irrespective of their potential side effects [10, 30]. Currently, concomitant use of vasoactive agents during mechanical LV unloading in CS is based on expert consensus and local practice. Norepinephrine is recommended as the first-line therapy if perfusion pressure is low [31], mainly based on the Sepsis Occurrence in Acutely Ill Patients (SOAP-II) trial. The SOAP-II trial demonstrated a lower risk of arrhythmia with norepinephrine among patients with shock. Moreover, norepinephrine was associated with improved survival compared to dopamine in a subgroup analysis of 280 patients with CS [32]. Avoidance of arrhythmia is pivotal in patients treated with the microaxial flow pump, given their functional dependence on adequate blood delivery from the right heart (preload). A recent randomized study that compared norepinephrine and epinephrine in 57 patients with AMICS demonstrated similar effects on perfusion pressure, but in the epinephrine group, a higher incidence of prolonged lactate-acidosis, tachycardia, and refractory CS was observed, leading to premature termination of the study [33]. In the present study, we did not observe any adverse metabolic effects of epinephrine, which may be a result of dosage or duration of therapy. However, we observed tachycardia with epinephrine as well as for dopamine and phenylephrine, which is concerning both in terms of adequate coronary perfusion and risk of arrhythmia.

The increase in HR with phenylephrine is intriguing, and current study offers no direct insight in the reason for this. The increase was not driven by one or two outliers or due to cardiac arrhythmias. Rather, we observed a uniform increase in HR. Whether this was caused by reflex tachycardia due to reduction in perfusion (reduction in SvO2) or whether it was a direct effect of the drug in Danish landrace pigs is speculative. Compared to dopamine that also caused significant increase in HR, the effect of dopamine was associated with increase in SvO2 whereas phenylephrine was not, suggesting the unfavorable effect of phenylephrine not solely to be driven by HR. Thus, the current study warrants caution to use of vasoconstrictor alone while on LV support with Impella CP in AMICS.

### Limitations

In this study, vasoactive drugs were administered at what is considered equipotent doses and not titrated against a predefined target MAP, as done in the clinical setting. The doses of dopamine and phenylephrine used in this study might have been too low compared to the new vasoactive inotropic score [34]. In our opinion, a higher dose of phenylephrine would not be beneficial given the adverse effect of the low dose used in this study. Also, increasing the dopamine dose would result in vasoconstriction (alpha-receptor stimulation) with the risk of negative impact on cardiac work and coronary perfusion. The experimental setting based on a standard operating procedure used in this study aids in reproducibility and reducing the variability involved in testing the physiological effects of drug therapies in an acute setting. A high number of animals would have been required to compare the effects inter-individual. Despite being clearly superior, this was not feasible in terms of time required and expenses. Thus, we chose to do the cross-over design and make intra-individual comparisons to allow for a lower number of animals. However, the small sample size of the study is a limitation and may result in a type II error. Given the risk of hemodynamic instability, we did not include washout periods between drug infusions. Nonetheless, we do not expect a carryover effect of a previous drug infusion as the vasoactive agents have a short half-life, and the measurements were taken at the end of each infusion. The diuresis was not recorded systematically as we in design of study considered the individual duration of intervention too short to have confidence that the diuresis during each intervention was not mostly carry over effect of previous. The crossover design and statistical analyses were undertaken considering the potential effect of the timing of the interventions. Phenylephrine was administered in all the pigs at the end of the experiment due to its long half-life. The pigs may have developed more severe CS in the end, which could have affected the results. We attempted to adjust for this effect by using the linear mixed model and believe that the adverse effect of phenylephrine observed in this study reflects the drug’s effect and not a time-dependent artifact. The experimental observation period in this study is probably too short and may not reflect the long-term effects of concomitant vasoactive treatment and LV unloading in AMICS. Given the similar body size and adrenoceptor distribution and function among pigs and humans, the results may apply to the human treatment of CS [24].

## Conclusion

In this preclinical study, mechanical circulatory support with Impella CP in severe CS lowered cardiac workload, but perfusion pressure was inadequate in most pigs. The addition of catecholamines increased perfusion pressure and oxygen delivery but at the expense of increased cardiac work, most for dopamine. Vasoconstriction with phenylephrine caused an increase in cardiac work without any increase in oxygen delivery. Thus, a support strategy based on Impella CP and low-dose catecholamine preferably norepinephrine to avoid arrhythmias seems optimal to balance oxygen delivery and cardiac work. The study results suggest great caution when using vasoconstrictors such as phenylephrine in the setting of CS.

## Data Availability

The datasets from the current study are available from the corresponding author on reasonable request.

## Abbreviations

AMI: Acute myocardial infarction
AMICS: Acute myocardial infarction complicated by cardiogenic shock
CI: Confidence interval
CO: Cardiac output
CS: Cardiogenic shock
Ea: Arterial elastance
Ees: End-systolic pressure-volume relationship
HR: Heart rate
LV: Left ventricle
LVEDP: Left ventricular end-diastolic pressure
LVEDV: Left ventricular end-diastolic volume
LVESP: Left ventricular end-systolic pressure
LVESV: Left ventricular end-systolic volume
LLM: Linear mixed model
MAP: Mean arterial blood pressure
PE: Potential energy
PV: Pressure volume
PVA: Pressure volume area
SvO_2_: Mixed venous oxygen saturation
SW: Stroke work
